# 618. The current state of laboratory mycology in Asia/Pacific: a survey from the EuropeanConfederation of Medical Mycology (ECMM) and International Society for Human andAnimal Mycology (ISHAM)

**DOI:** 10.1093/ofid/ofad500.684

**Published:** 2023-11-27

**Authors:** Jon Salmanton-Garcia, Au Wing-Yan, Martin Hoenigl, Arunaloke Chakrabarti, Oliver A Cornely

**Affiliations:** University Hospital Cologne, Cologne, Germany, Cologne, Nordrhein-Westfalen, Germany; Med Clinic, Central, Hong Kong, Hong Kong, Not Applicable, Hong Kong; Medical University of Graz, Department of Internal Medicine, Division of Infectious Diseases, Graz, Steiermark, Austria; 19Department of Medical Microbiology, Postgraduate Institute of Medical Education and Research, Chandigarh, Chandigarh, Chandigarh, India; University of Cologne, Cologne, Germany, Cologne, Nordrhein-Westfalen, Germany

## Abstract

**Background:**

Invasive fungal infections (IFI) in Asia/Pacific are a threat to patients with malignancies, uncontrolled diabetes mellitus or undiagnosed/untreated human immunodeficiency virus infection and acquired immunodeficiency syndrome (HIV/AIDS). Adequate and early access to diagnostic tools and antifungals is essential for the IFI clinical management and patient survival.

**Methods:**

The IFI diagnostic capacity survey was online accessible at www.clinicalsurveys.net/uc/IFI_management_capacity/. The survey aimed to collect the following dimensions: a) institution profile, b) perceptions on IFI in the respective institution, c) microscopy, d) culture and fungal identification capacity, e) serology, f) antigen detection, g) molecular tests availability, and h) therapeutic drug monitoring.

**Results:**

As of June 2022, 235 centres from 40 countries/territories in Asia/Pacific answered the questionnaire. More than half of them were from six countries: India (25%), China (17%), Thailand (5%), Indonesia, Iran, and Japan (4%, each). Candida spp. (93%) and Aspergillus spp. (75%) were considered the most relevant pathogens. Most institutions had access to microscopy (98%) or culture-based approaches (97%). Furthermore, 79% of them had access to antigen detection, 66% to molecular assays, and 63% to antibody tests. Access to antifungals varied between countries/territories. At least one triazole was available in 93% of the reporting sites (voriconazole [89%] was the most common mould-active azole), whereas 80% had at least one amphotericin B formulation, and 72% at least one echinocandin.

**Conclusion:**

Currently, based on the provided replies, the resources available for the IFI diagnosis and management vary in Asia/Pacific countries/territories. Economical or geographical factors may play a key role in the incidence and clinical handling of this disease burden. Regional cooperation may be a good strategy to overcome shortcomings.

**Disclosures:**

**Martin Hoenigl, n/a**, Astellas: Grant/Research Support|Euroimmun: Grant/Research Support|F2G: Grant/Research Support|Gilead: Grant/Research Support|Immy: Grant/Research Support|MSD: Grant/Research Support|Mundipharma: Grant/Research Support|Pfizer: Grant/Research Support|Pulmocide: Grant/Research Support|Scynexis: Grant/Research Support **Oliver A. Cornely, MD PhD**, DZIF: Advisor/Consultant|DZIF: Board Member|DZIF: Grant/Research Support|DZIF: Honoraria|DZIF: Stocks/Bonds

